# A CNN-GRU Fusion Mathematical Model for Positioning Jump Correction in Integrated Navigation Systems

**DOI:** 10.3390/s26175370

**Published:** 2026-08-25

**Authors:** Mingyang Deng, Guangjiao Chen

**Affiliations:** 1 College of Civil Engineering and Transportation, Beihua University, Jilin 132013, China; dengmy@beihua.edu.cn; 2 Vehicle-Road-Cloud Integrated Innovation Platform, Beihua University, Jilin 132013, China

**Keywords:** CNN-GRU, integrated navigation, positioning jump correction, mathematical model, urban canyon

## Abstract

Positioning jumps are a primary cause of trajectory discontinuities in urban canyon environments, severely hindering the widespread adoption of autonomous vehicles. This paper proposes a CNN-GRU fusion-based method for correcting such positioning jumps. A complete 15-dimensional error-state extended Kalman filter (EKF) framework is established to analyze the jump generation mechanisms from three perspectives—pseudorange distortion, inertial drift, and filter gain divergence—thereby justifying the use of inertial measurement unit (IMU) time-series data for anomaly prediction. An end-to-end mapping model is further constructed, in which a one-dimensional convolutional neural network (CNN) extracts cross-channel spatial features from multi-axis inertial data, while a gated recurrent unit (GRU) captures long-term temporal error evolution. A hysteresis navigation quality factor and a piecewise Huber loss function are incorporated to enable hierarchical adaptive optimization. Experimental results demonstrate that the proposed method reduces the positioning root mean square error (RMSE) from 1.24 m to 0.70 m, achieves a jump suppression rate of 43.5%, and maintains a single-frame inference latency of 11.8 ms, meeting the real-time requirements for future autonomous driving localization.

## 1. Introduction

High-level autonomous driving systems demand continuous, high-precision, and robust lane-level localization to underpin environmental perception, path planning, and vehicle motion control [1]. The GNSS/INS integrated navigation system, which synergistically combines the long-term absolute accuracy of GNSS with the high-rate continuous output of inertial measurement units (IMUs), has become the de facto positioning solution for mass-produced intelligent vehicles. However, in typical urban canyons, under viaducts, and in dense foliage, line-of-sight (LOS) satellite signals are severely attenuated, and multipath reflections substantially distort pseudorange observations [2]. These effects induce instantaneous meter-level positioning jumps and step-like trajectory discontinuities, which in turn cause oscillations in path planning and instability in lateral control, thereby posing a serious threat to autonomous driving safety [3].

Conventional approaches to mitigate localization errors fall into three main categories. The first category comprises fixed-gain Kalman filters and their adaptive variants, which adjust the noise covariance matrix based on residual statistics [4,5]. Nevertheless, such methods offer only limited improvement for slowly varying noise and exhibit pronounced lag when responding to abrupt non-line-of-sight (NLOS) outliers, failing to eliminate step-type jumps fundamentally. The second category leverages additional sensors such as LiDAR and cameras in multi-sensor fusion frameworks; while these schemes enhance robustness, their high hardware cost and computational burden impede wide deployment in low-cost vehicle terminals [6,7]. The third category, driven by recent advances in deep learning, employs temporal neural networks for navigation error prediction and compensation [8,9,10]. Convolutional neural networks (CNNs) are particularly effective at extracting spatial coupling features from multi-channel sensor data, whereas recurrent neural networks (RNNs), including gated recurrent units (GRUs), excel at modeling long-term temporal dependencies [11].

Despite these efforts, existing CNN-GRU integrated navigation models are pre-dominantly designed for error compensation under complete GNSS outages, with little systematic attention paid to the localization jump problem caused by partial signal degradation [12]. Moreover, most deep learning-based navigation models lack rigorous mathematical derivation and interpretability [13]. In addition, the common practice of using a single loss function cannot adaptively discriminate between severe abnormal outliers and mild trajectory jitters, often resulting in either insufficient jump suppression or excessive smoothing distortion [14].

This paper proposes a hierarchical adaptive model fusing a 1D CNN with a modified GRU for positioning jump correction. Theoretically, GNSS NLOS errors propagate through the EKF and leave traces in the error-state evolution. Section 2.3 shows that the posterior estimation error depends on both GNSS and IMU noises, implying that IMU-state consistency encodes predictive information for anomalies. This motivates a deep architecture to extract IMU spatial features and map them to EKF corrections.

The main contributions are as follows. First, we derive a 15-D error-state EKF to analyze three mechanisms of NLOS-induced jumps, establishing the theoretical basis for IMU prediction. Second, we propose a modified GRU with adaptive gated scaling for enhanced long-term memory, and construct an interpretable spatio-temporal fusion framework to capture IMU spatial features and error temporal evolution. Third, we introduce a hysteresis quality factor for adaptive hierarchical discrimination and design a piecewise Huber loss to balance robustness and fidelity by differentially handling outliers and jitters. Fourth, we perform vehicle experiments and ablation studies on urban canyon data, with MATLAB analysis, to validate accuracy, jump suppression, and re-al-time performance. The remainder is organized as follows. Section 2 presents GNSS/INS fundamentals and jump generation. Section 3 details the proposed 1D-CNN/mGRU model. Section 4 describes experiments and results. Section 5 concludes and discusses future work.

## 2. Navigation Fundamentals and Localization Jump Mechanism

The research demonstrates that IMU time-series data can provide a motion baseline, propagate GNSS anomalies into detectable deviations, and enable data-driven mapping of these deviations. Therefore, leveraging the mechanism of predicting EKF localization errors from IMU time-series data, this paper proposes a correction model architecture. The network is specifically designed to: (a) extract spatial features from multi-channel IMU data via a 1D CNN; (b) model the temporal evolution of localization errors using a modified GRU; and (c) perform hierarchical adaptive correction under the guidance of a hysteresis quality factor.

### 2.1. 15-Dimensional Error-State EKF Model

The loosely coupled GNSS/INS integrated navigation architecture is adopted, and a 15-dimensional error state vector is constructed to comprehensively describe position error, velocity error, attitude misalignment angle, gyroscope drift, and accelerometer bias. The state vector is defined as [15]:

(1) $$\begin{array}{c} x={\left[\delta p^{T},\delta v^{T},\phi^{T},b_{g}^{T},b_{a}^{T}\right]}^{T}\in R^{15} \end{array}$$

where $\delta p\in R^{3}$ and $\delta v\in R^{3}$ denote the three-dimensional position error and velocity error in the navigation coordinate system, respectively; $\phi \in R^{3}$ represents the attitude misalignment angle vector; $b_{g}\in R^{3}$ and $b_{a}\in R^{3}$ are the drift vector of the gyroscope and the bias vector of the accelerometer, respectively.

(1) State Prediction Equation

The discrete state prediction process of EKF is expressed as [15]:

(2) $$\begin{array}{c} {\hat{x}}_{k|k-1}=F_{k}{\hat{x}}_{k-1|k-1}+w_{k},w_{k}∼N\left(0,Q_{k}\right) \end{array}$$

(3) $$\begin{array}{c} P_{k|k-1}=F_{k}P_{k-1|k-1}F_{k}^{T}+Q_{k} \end{array}$$

where $F_{k}$ is the time-varying state transition matrix; $w_{k}$ denotes the system process noise vector obeying Gaussian distribution; $Q_{k}$ represents the process noise covariance matrix; $P_{k|k-1}$ is the prior state estimation covariance matrix.

(2) GNSS Observation Equation

The GNSS position and velocity observation equations are constructed as follows [15]:

(4) $$\begin{array}{c} z_{k}=H_{k}x_{k}+v_{k},v_{k}∼N\left(0,R_{k}\right) \end{array}$$

where $z_{k}$ is the GNSS observation vector; $H_{k}$ denotes the observation mapping matrix; $v_{k}$ represents the Gaussian observation noise vector; $R_{k}$ is the observation noise covariance matrix.

(3) Filter Update Equation

The Kalman gain calculation, posterior state estimation, and covariance update formulas are given by [15,16]:

(5) $$\begin{array}{c} K_{k}=P_{k|k-1}H_{k}^{T}{\left(H_{k}P_{k|k-1}H_{k}^{T}+R_{k}\right)}^{-1} \end{array}$$

(6) $$\begin{array}{c} {\hat{x}}_{K|k}={\hat{x}}_{k|k-1}+K_{k}\left(z_{k}-H_{k}{\hat{x}}_{k|k-1}\right) \end{array}$$

(7) $$\begin{array}{c} P_{k|k}=\left(I-K_{k}H_{k}\right)P_{k|k-1} \end{array}$$

Traditional EKF adopts a fixed $R_{k}$ matrix, which cannot adapt to the sharp degradation of GNSS signal quality in NLOS scenarios. The mismatch of noise statistical characteristics leads to excessive Kalman gain, amplifies abnormal observation errors, and eventually causes severe localization jumps.

### 2.2. Definition and Generation Mechanism of Localization Jump

(1) Quantitative Criterion of Localization Jump

To accurately identify abnormal localization jumps, the 2-norm of the inter-frame planar position deviation is defined as the jump amplitude evaluation index [16]:

(8) $$\begin{array}{c} \Delta d_{k}=\|{\hat{P}}_{k|k}-{\hat{P}}_{k-1|k-1}{\|}^{2} \end{array}$$

Combined with vehicle kinematic physical constraints, the maximum feasible single-frame displacement satisfies the following condition:

(9) $$\begin{array}{c} \Delta d_{k}\leq v_{max}\Delta t+\frac{1}{2}a_{max}\Delta t^{2} \end{array}$$

where the maximum vehicle speed $v_{max}=15\;\mathrm{km}/h$, the maximum acceleration $a_{max}=3\;m/s^{2}$, and the system sampling interval Δt=0.1 s. If the actual inter-frame displacement fails to satisfy Equations (8) and (9), the current epoch is judged as a localization jump outlier without physical motion support.

(2) Three Mathematical Causes of Localization Jump

(a) Multipath pseudorange distortion: The ideal geometric pseudorange observation model of GNSS satellites is [16]:

(10) $$\begin{array}{c} \rho_{i}=r_{i}+c\left(\delta t_{u}-\delta t_{s}\right)+I_{i}+T_{i}+\epsilon_{\rho } \end{array}$$

where $r_{i}$ is the geometric distance between the satellite and the receiver; c denotes the speed of light; $\delta t_{u}$ and $\delta t_{s}$ represent the receiver clock error and satellite clock error, respectively; $I_{i}$ and $T_{i}$ are the ionospheric delay and tropospheric delay; $\epsilon_{\rho }$ denotes random noise. In NLOS scenarios, reflection interference introduces an extra deviation $\Delta \rho_{\mathrm{bias}}$, forming distorted pseudorange ${\hat{\rho }}_{i}=\rho_{i}+\Delta \rho_{\mathrm{bias}}$ and directly causing meter-level coordinate mutation.

(b) Discontinuous inertial drift compensation: Under long-term GNSS outage conditions, the accelerometer bias continuously accumulates position errors through double integration:

(11) $$\begin{array}{c} \Delta p_{drift}=\iint C_{b}^{n}b_{a}dt^{2} \end{array}$$

where $C_{b}^{n}$ is the direction cosine matrix from the body coordinate system to the navigation coordinate system. When satellite signals recover suddenly, the filter compensates for all accumulated drift errors at one time, resulting in discontinuous trajectory steps.

(c) Kalman gain divergence: The fixed observation noise matrix cannot adapt to the sharp increase in residuals in NLOS scenarios. The excessive filter gain makes distorted GNSS observations dominate the state update, further amplifying localization oscillation and jump distortion.

Although GNSS NLOS errors are not directly observable from IMU measurements, the resulting localization jumps are manifested as discrepancies between IMU-predicted states and GNSS observations. Specifically, the innovation vector ${\tilde{y}}_{k}=z_{k}-H_{k}\;{\hat{x}}_{k|k-1}$ becomes statistically abnormal during NLOS periods. Since this innovation anomaly correlates with the IMU temporal sequence (through the prediction step), the IMU data implicitly encode the information needed to predict jumps. This theoretical insight motivates our end-to-end learning framework: the network learns the mapping from IMU patterns to correction vectors without explicit GNSS NLOS detection.

### 2.3. Theoretical Basis for IMU-Based Prediction of Localization Jumps

A critical question arises: since GNSS NLOS errors originate from satellite signal blockage, how can a model relying primarily on IMU measurements predict the resulting localization jumps? This subsection establishes the theoretical foundation for the feasibility of IMU-based jump prediction from three complementary perspectives.

(1) Kinematic Constraint Transitivity

Under NLOS conditions, GNSS pseudorange observations contain large positive biases, causing the EKF to generate position estimates that violate the vehicle’s physical motion constraints. Specifically, the true vehicle motion must satisfy [15,16,17]:

(12) $$\|P_{k}-P_{k-1}{\|}^{2}\leq v_{\max}\cdot \Delta t+\frac{1}{2}a_{\max}\Delta t^{2}$$

where $v_{max}$ and $a_{max}$ and are the maximum feasible velocity and acceleration given the vehicle’s dynamic envelope. When GNSS observations are corrupted by NLOS errors, the EKF output violates this inequality, producing a “jump” pattern in the position trajectory. Crucially, the IMU measurements, which are insensitive to GNSS signal quality, provide a continuous and physically consistent representation of the vehicle’s actual motion. The discrepancy between the EKF-estimated motion (contaminated by NLOS) and the IMU-indicated motion (physically plausible) thus serves as a detectable signature of GNSS anomalies.

(2) Error-State Temporal Propagation

To formalize the relationship between IMU measurements and EKF output errors, we analyze the error-state propagation mechanism. From the EKF prediction and update equations, the posterior state estimation error can be rigorously derived as [16,17]:

(13) $${\tilde{x}}_{k|k}=\left(I-K_{k}H_{k}\right)F_{k}{\tilde{x}}_{k-1|k-1}+\left(I-K_{k}H_{k}\right)w_{k-1}-K_{k}v_{k}$$

where $w_{k-1}∼N(0,Q_{k-1})$ denotes the IMU process noise (primarily gyroscope drift and accelerometer bias), and $v_{k}∼N(0,R_{k})$ denotes the GNSS observation noise, which becomes abnormally large under NLOS conditions. This equation reveals a crucial property: the GNSS observation noise $v_{k}$ does not directly appear in the IMU measurements, but its effect propagates through the Kalman gain $K_{k}$ and contaminates the entire state vector. In other words, the NLOS-induced error leaves detectable “traces” in the state evolution pattern—traces that manifest as deviations from the expected IMU-state consistency.

The IMU measurements $U_{k}=[\omega_{k},a_{k}]$ provide a reference baseline for what the state evolution should be in the absence of GNSS corruption. By observing the temporal sequence of IMU data within a sliding window $U_{k}$, the network learns to identify these deviation patterns and predict the corresponding correction ${\hat{\delta }}^{k}$. This is mathematically feasible because the mapping from IMU features to EKF errors is a continuous function (albeit highly nonlinear) that can be approximated by the proposed 1D-CNN/mGRU architecture.

(3) Data-Driven Functional Approximation

It is important to clarify that the network does not attempt to “infer” the physical NLOS conditions from IMU data that a physically underdetermined inverse problem. Instead, the network learns a data-driven approximation of the complex nonlinear relationship [17,18]:

(14) $$\delta_{k}=F\left(U_{k};\theta \right)+\epsilon_{k}$$

where F is an unknown but deterministic function mapping the recent history of IMU measurements to the EKF output error, and $\epsilon_{k}$ represents the irreducible approximation residual. During training, the network observes a large number of examples $\left\{U_{k},\delta_{k}^{true}\right\}$ collected across diverse urban NLOS scenarios. Through supervised learning, it identifies the statistical regularities between IMU-derived motion features and EKF error patterns—essentially learning a surrogate model for the complex interaction between vehicle dynamics and GNSS error propagation. This approach is conceptually aligned with residual learning [17,18], where the network learns to predict the residual between the corrupted estimate and the ground truth.

### 2.4. Predictability Analysis of Localization Jump

Urban localization jumps are not purely random noise, and the spatio-temporal characteristics of IMU data can support the prediction and correction of localization anomalies. The theoretical basis is summarized as follows. First, inertial navigation errors satisfy Lipschitz continuity, and the drift evolution is smooth in a short-time window, ensuring traceable error variation. Second, satellite signal quality degrades gradually in urban occlusion scenarios, providing an effective early warning window for jump prediction. Third, vehicle motion states such as turning and bumping are strongly coupled with multipath interference intensity, which can be fully characterized by six-axis IMU observations. Fourth, urban occlusion scenarios exhibit spatial repeatability, enabling the network to learn typical temporal laws of localization jumps.

## 3. Proposed 1D-Cnn and Modified GRU Hierarchical Correction Model

### 3.1. Temporal Regression Task Modeling

The single-epoch six-axis IMU observation vector is constructed as $u_{k}={\left[\omega_{k}^{T},a_{k}^{T}\right]}^{T}\in R^{6}$, where $\omega_{k}$ and $a_{k}$ denote angular velocity and specific force observations, respectively. A sliding time window with length T=50 is adopted to construct the network input matrix [19]:

(15) $$\begin{array}{c} U_{k}=\left[U_{k-T+1},U_{k-T+2},\ldots ,U_{k}\right]\in R^{T\times 6} \end{array}$$

The sliding window length is set to T=50 corresponding to 50 × 0.1 s = 5 s of historical IMU data at the 10 Hz sampling rate. This value is selected through a systematic ablation study over $T\in \left\{10,20,30,50,80,100\right\}$ on the validation set. The results show that T=50 achieves the optimal balance between prediction accuracy and computational latency: shorter windows T≤30 fail to capture sufficient temporal context for low-frequency navigation error evolution, while longer windows T≥80 introduce redundant information and increased inference latency without commensurate accuracy gains.

The end-to-end nonlinear mapping relationship of the proposed model is defined as [19]:

(16) $$\begin{array}{c} {\hat{\delta }}_{k}=M_{\theta }\left(U_{k};\Theta \right) \end{array}$$

where ${\hat{\delta }}_{k}$ is the predicted localization correction vector; $M_{\theta }\left(\cdot \right)$ denotes the mapping function of the proposed network; Θ represents all trainable parameters of the network. The optimal parameter set is obtained by minimizing the loss function [19]:

(17) $$\begin{array}{c} \Theta^{*}=arg\;\underset{\Theta }{min}\frac{1}{N}\overset{N}{\underset{k=1}{\sum L}}\left(M_{\theta }\left(U_{k};\Theta \right),\delta_{k}^{true}\right) \end{array}$$

where $\delta_{k}^{\mathrm{true}}$ is the real localization error solved by high-precision real-time kinematic (RTK) ground truth; N denotes the total number of samples; $L\left(\cdot \right)$ is the custom loss function.

### 3.2. 1D-CNN Spatial Feature Extraction

IMU measurements comprise six-axis synchronized signals with inherent cross-channel coupling. 1D-CNN is adopted over 2D-CNN or Transformer for two reasons: (i) the temporal dimension of IMU data exhibits local stationarity, making the local receptive field of 1D-CNN physically interpretable as capturing short-term motion patterns; (ii) the computational complexity of 1D-CNN is O(T·C·K), significantly lower than 2D-CNN O(T·C·H·W) and attention-based O(T^2^·C), which is critical for onboard real-time deployment.

A two-layer 1D-CNN is adopted to extract cross-channel spatial coupling features of multi-sensor IMU data. For the input temporal matrix $U\in R^{T\times C}$ (C=6), the convolution calculation is expressed as [20]:

(18) $$\begin{array}{c} Z_{i,f}=\sigma \left(\sum_{c=1}^{C}\sum_{l=0}^{L-1}U_{i+l,c}\cdot W_{l,c,f}+b_{f}\right) \end{array}$$

where the activation function $\sigma \left(\cdot \right)=\mathrm{ReLU}$; $W_{l,c,f}$ and $b_{f}$ denote the convolution kernel weight and bias, respectively. The first convolution layer adopts a kernel length of 5 and 64 feature channels, while the second convolution layer adopts a kernel length of 3 and 128 feature channels. Batch normalization and max-pooling operations are embedded to eliminate redundant features and enhance spatial feature representation capability.

The 1D-CNN architecture is designed with two convolutional layers. The first layer adopts a kernel length of 5 and 64 feature channels, while the second layer adopts a kernel length of 3 and 128 feature channels. These hyperparameters are selected via a grid search on the validation set, with kernel lengths searched over (3,5,7) and channel numbers searched over (32,64,128,256). The optimal configuration—kernel lengths (5,3) and channels (64,128)—achieves the best trade-off between feature extraction capability and computational efficiency. The larger kernel in the first layer captures short-term motion patterns, while the smaller kernel in the second layer extracts finer spatial features from the intermediate representations. Batch normalization and max-pooling operations are embedded after each convolutional layer to eliminate redundant features and enhance spatial feature representation capability.

### 3.3. Modified GRU Temporal Memory Unit

Traditional GRU lacks sufficient long-term temporal feature retention capability for low-frequency, slowly varying navigation errors. In this paper, an adaptive scaling coefficient is introduced to optimize the update gate structure, improving the long-sequence error modeling performance. The complete formulas of the modified GRU are presented as follows.

Standard GRU suffers from rapid information decay when processing low-frequency navigation errors. The update gate $u_{t}$ determines the retention of historical states. However, under slowly varying GNSS degradation, the error evolves as a low-frequency perturbation, yet the standard update gate remains overly sensitive to instantaneous inputs, leading to premature state refresh. To address this, we introduce a scaling coefficient γ = 0.8 to compress the update gate activation, yielding slower state evolution and enhanced long-term memory retention.

Reset gate:

(19) $$\begin{array}{c} r_{t}=\sigma \left(W_{r}z_{t}+U_{r}h_{t-1}+b_{r}\right) \end{array}$$

where γ is the scaling coefficient, set to 0.8. γ=0.8 is selected based on grid search. The optimal γ lies in [0.75, 0.85], corresponding to the typical NLOS error correlation time constant of 5–10 s. This alignment indicates that the scaling coefficient effectively matches the temporal characteristics of urban GNSS degradation.

The scaling coefficient γ=0.8 is introduced to suppress the update gate, thereby enhancing the long-term memory retention of the GRU. The value is determined through a grid search over $\gamma \in \left\{0.5,0.6,0.7,0.8,0.9,1.0\right\}$ on the validation set, with γ=0.8 yielding the minimum validation RMSE. Theoretically, when γ<1, the effective memory time constant is $\tau \approx 1/\left(1-\gamma \right)=5$ time steps, which aligns with the typical correlation time scale of IMU drift errors (approximately 0.5 s at 10 Hz).

(20) $$\begin{array}{c} u_{t}=\sigma \left(\gamma \cdot \left(W_{u}z_{t}+U_{u}h_{t-1}+b_{u}\right)\right) \end{array}$$

Candidate hidden state:

(21) $$\begin{array}{c} {\tilde{h}}_{t}=tanh\left(W_{h}z_{t}+U_{h}\left(r_{t}⊙h_{t-1}\right)+b_{h}\right) \end{array}$$

Output hidden state:

(22) $$\begin{array}{c} h_{t}=\left(1-u_{t}\right)⊙h_{t-1}+u_{t}⊙{\tilde{h}}_{t} \end{array}$$

where ⊙ denotes the Hadamard product; $z_{t}$ is the spatial feature vector extracted by the CNN. Two layers of modified GRU are stacked with 64 and 32 hidden units, respectively, balancing temporal fitting accuracy and embedded computational efficiency.

### 3.4. Hysteresis Hierarchical Correction Strategy

To adaptively distinguish strong and weak interference working conditions, a dimensionless navigation quality factor is constructed by integrating jump amplitude, position dilution of precision (PDOP), and the number of valid satellites:

(23) $$\begin{array}{c} Q_{k}=\alpha {\tilde{Q}}_{k}+(1-\alpha )Q_{k\text{-}1} \end{array}$$

where α∈(0,1) is the hysteresis smoothing factor that controls the temporal persistence of the quality evaluation, preventing frequent mode switching due to transient fluctuations. $Q_{k\text{-}1}$ is the quality factor from the previous time step. $Q_{k}$ denotes the hysteresis navigation quality evaluation factor at time step k, which integrates both instantaneous and historical navigation quality indicators; ${\tilde{Q}}_{k}$ is the instantaneous quality indicator computed from current-frame measurements, defined as:

(24) $$\begin{array}{c} Q_{k}=\omega_{1}\frac{\Delta d_{k}}{\Delta d_{th}}+\omega_{2}\frac{{PDOP}_{k}}{{PDOP}_{th}}+\omega_{3}\frac{N_{th}-N_{sat,k}}{N_{th}} \end{array}$$

where the weight coefficients $\omega_{1}=0.4,\omega_{2}=0.3,\omega_{3}=0.3$; $\Delta d_{K}$ being the single-frame displacement, the threshold parameters $\Delta d_{th}=1.0\;m$, ${PDOP}_{th}=2.0$, and $N_{th}=6$. To avoid frequent working condition switching and oscillation, a hysteresis switching mechanism is designed. If $Q_{k}\leq 0.3$ for five consecutive frames, the working condition is judged as stable and superior, and the basic correction strategy is adopted. If $Q_{k}\geq 0.7$ for two consecutive frames, the working condition is judged as a severe NLOS condition, and the adaptive attenuation correction coefficient is introduced:

(25) $$\begin{array}{c} \eta_{k}=exp\left(-\lambda \cdot \frac{\Delta d_{k}}{\Delta d_{th}}\right) \end{array}$$

The final correction output of the model is ${\hat{\delta }}_{k}=M_{\mathrm{coarse}}\left(U_{k}\right)\cdot \eta_{k}+\delta_{k}^{\mathrm{INS}}$. For the transition interval $0.3<Q_{k}<0.7$, the current working condition is locked for 3 s to ensure model operation stability. The parameter γ=0.8 controls the update rate of the hysteresis factor $Q_{k}$, balancing the responsiveness to sudden quality degradation and the stability against short-term noise fluctuations. A larger λ (closer to 1) emphasizes historical quality, yielding smoother but more lagging corrections; a smaller λ emphasizes instantaneous quality, enabling faster response but increasing the risk of false triggers due to temporary GNSS outliers. The value γ=0.8 is selected empirically based on preliminary experiments and validated through sensitivity analysis, which shows that $\lambda \in \left[0.7,0.9\right]$ yields stable performance with less than 3% variation in RMSE.

### 3.5. Piecewise Huber Robust Loss Function

To balance the suppression of large-scale abnormal outliers and the fitting accuracy of tiny trajectory jitters, a piecewise Huber loss function is adopted for model training [21]:

(26) $$\begin{array}{c} L_{\mathrm{Huber}}\left(\delta ,\hat{\delta }\right)=\left\{\begin{array}{c} \frac{1}{2}∥\delta -\hat{\delta }{∥}_{2}^{2},∥\delta -\hat{\delta }{∥}_{1}\leq \beta \\ \beta ∥\delta -\hat{\delta }{∥}_{1}-\frac{1}{2}\beta^{2},otherwise \end{array}\right. \end{array}$$

where the hyperparameter β=0.5. The dataset is divided into a training set and a test set at a ratio of 7:3. The Adam optimizer is adopted with an initial learning rate of $10^{-3}$ and a batch size of 128. An early stopping strategy is utilized to prevent model overfitting. $e={\hat{\delta }}_{k}-\delta_{k}^{true}$ is the prediction residual; the threshold δ is set to δ=1.345 MAE, following the standard recommendation for robust regression [21], where MAE is the mean absolute error of the predictions on the validation set. This choice ensures that approximately 95% of the residuals are treated quadratically (smooth optimization for small errors), while the remaining 5% with larger residuals are handled linearly (reducing the influence of outliers). The threshold is adaptively determined from the validation data rather than being fixed arbitrarily.

### 3.6. Proposed Algorithm Procedure

To standardize the model implementation and conform to IEEE journal specifications, the overall algorithm flow of the proposed model is presented in Algorithm 1.
**Algorithm 1:** IMU/GNSS Fusion Localization with Trajectory Jump SuppressionNotations: ***U_k_***: IMU sliding window matrix; ***Z_k_***: spatial feature vector; ***h****_t_*: hidden state; **Δ*d_k_***: inter-frame jump; ***Q_k_***: navigation quality factor; $\hat{\delta_{k}}$: corrected positioning error Hyperparameters: ***T*** = 50, ***γ*** = 0.8, ***β*** = 0.5, ***ω***_1_ = 0.4, ***ω***_2_ = 0.3, ***ω***_3_ = 0.3, Δ***d_th_*** = 1.0 m, ***PDOP_th_*** = 2.0, ***N_th_*** = 6 Input: Six-axis IMU time-series data, GNSS localization observations; Output: Jump-suppressed high-precision continuous vehicle localization trajectory; Normalize IMU, build ***U_k_*** ∈ ***R***^(***T*** × 6), init ***h*_0_** and network params; ***Z_k_*** = CNN_Block(***U_k_****)* (2-layer 1D-CNN: Conv, BN, MaxPool); for ***t*** = 1, …, ***T***        ***r_t_*** =***σ***(***W_r_ z_t_*** +***U_r_******h_t_*_-1_** + ***b_r_***);        ***u_t_*** =***σ***(***γ***(***W_u_ z_t_*** +***U_u_******h_t_*_-1_** + ***b****_u_*));        $\tilde{h_{t}}$ = **tanh**(***W_h_ z_t_*** + ***U_h_*(*r_t_***⊙***h_t_*_-1_**) + ***b_h_***);        ***h_t_*** = (1 − ***u_t_***)⊙***h_t_***_-1_ + ***U_t_***⊙$\tilde{h_{t}}$; end for Compute Δ***d_k_***, ***PDOP***, ***N_sa_*_t_**, ***Q_k_***;        if ***Q_k_*** ≤ 0.3 for 5 frames then            Apply adaptive attenuation correction;                else if ***Q_k_*** ≥ 0.7 for 2 frames then                     else: Lock current condition;                 else if Fuse errors: $\hat{\delta_{k}}$ = ***M_coarse_***(***U_k_***)·***η_k_*** + ***δ_k_***^INS, suppress GNSS jump outliers;            Optimize with Huber loss and Adam, apply early stopping;        Return high-continuity and high-precision trajectory;End

## 4. Experimental Results and Analysis

### 4.1. Experimental Platform and Evaluation Metrics

The vehicle experimental platform is equipped with a 100 Hz micro-electro-mechanical system (MEMS) six-axis inertial measurement unit (IMU), a 10 Hz BeiDou/global navigation satellite system (GNSS) receiver, and a high-precision real-time kinematic (RTK) positioning system. The RTK system provides a horizontal positioning accuracy of ±0.1 m, which is regarded as the ground truth in this study. The total length of the test road is 4.5 km, covering typical urban non-line-of-sight (NLOS) scenarios such as high-rise building occlusion, viaduct shelter, and tree shade shielding. The vehicle driving speed varies from 20 km/h to 50 km/h, and a total of 72,000 valid time-series samples are collected in the field test. To fully verify the effectiveness and superiority of the proposed method, six algorithms are adopted for comparative experiments, including the raw GNSS positioning (R-GNSS), fixed-parameter extended Kalman filter (F-EKF), Sage–Husa adaptive extended Kalman filter (SH-EKF), adaptive extended Kalman filter (AEKF), single modified gated recurrent unit (S-GRU), and the proposed convolutional neural network-modified gated recurrent unit (CNN-GRU) hierarchical model [22,23,24,25,26].

Three quantitative indices, namely RMSE, maximum jump amplitude, and jump suppression rate, are adopted to comprehensively evaluate the model performance. The index definitions are as follows:

(27) $$\begin{array}{c} RMSE=\sqrt{\frac{1}{N}\overset{N}{\underset{k=1}{\sum }}\|{\hat{p}}_{k}-p_{k}^{\mathrm{RTK}}{\|}_{2}^{2}} \end{array}$$

(28) $$\begin{array}{c} \Delta d_{max}=\underset{1\leq k\leq N}{max}\Delta d_{k} \end{array}$$

(29) $$\begin{array}{c} \eta_{\sup}=\left(1-\frac{RM_{\mathrm{pro}}}{RM_{\mathrm{raw}}}\right)\times 100\% \end{array}$$

### 4.2. Quantitative Comparison Results

The quantitative performance comparison results of different algorithms in urban canyon NLOS scenarios are shown in Table 1. The raw GNSS suffers from severe abnormal jumps with a maximum jump amplitude of 4.72 m, which cannot meet the lane-level positioning requirements.

As shown in Table 1, the raw GNSS (R-GNSS) solution exhibits severe positioning jumps with an RMSE of 1.24 m and a maximum jump amplitude of 4.72 m, falling far short of lane-level navigation requirements. The fixed-gain EKF (F-EKF) provides moderate improvement, reducing the RMSE to 0.98 m and the maximum jump to 1.78 m, corresponding to a suppression rate of only 20.9%. The two adaptive filtering methods, SH-EKF and AEKF, achieve better performance, with RMSE values of 0.89 m and 0.87 m, and maximum jump amplitudes of 1.35 m and 1.21 m, respectively. Their suppression rates reach 28.2% and 29.8%, confirming the effectiveness of adaptive covariance adjustment for slowly varying noise. However, both methods exhibit a notable lag in responding to sudden NLOS anomalies due to the window-based accumulation of residual statistics. The standalone modified GRU (S-GRU) surpasses the adaptive EKF variants, attaining an RMSE of 0.85 m and a maximum jump of 0.91 m (suppression rate: 31.4%), yet its correction capability remains limited in sustained occlusion scenarios, primarily owing to the absence of spatial feature extraction.

By contrast, the proposed CNN-GRU model delivers the best overall performance across all metrics. It reduces the RMSE to 0.70 m, improves the suppression rate to 43.5%, and limits the maximum jump amplitude to 0.48 m—less than one-tenth of the raw GNSS value. The inference latency of 11.8 ms remains well below the 20 ms real-time constraint for embedded vehicle systems, confirming its practical deployability. The performance gains are attributable to two key design features: (i) the 1D-CNN module extracts cross-channel spatial coupling features from IMU data that are inaccessible to the GRU alone; and (ii) the hierarchical correction strategy with piecewise Huber loss enables differentiated optimization for severe outliers and minor trajectory jitters, a capability fundamentally absent in conventional EKF-based approaches.

It should be noted that although the RTK reference achieves ±0.1 m accuracy, the proposed method directly corrects the raw GNSS/INS loosely coupled solution. The final RMSE of 0.70 m thus represents the residual error after correction relative to the raw solution, not the RTK truth. Nevertheless, this level of accuracy satisfies the lateral lane-level requirement (<1.5 m) for the majority of autonomous driving applications.

### 4.3. Ablation Experiment Analysis

To quantitatively assess the individual contributions and complementary benefits of each network module, we conduct ablation experiments on three model variants [17,18,19]. The results are summarized in Table 2.

As presented in Table 2, the CNN+FC model, which relies exclusively on spatial feature extraction via convolutional layers without any temporal recurrence, yields an RMSE of 0.89 m and a maximum jump amplitude of 1.03 m. This result suggests that single-frame spatial features alone are inadequate for characterizing the temporal evolution of navigation errors. In comparison, the Single mGRU model, which performs temporal modeling in the absence of spatial feature extraction, achieves an improved RMSE of 0.85 m and reduces the maximum jump to 0.91 m, highlighting the critical role of temporal context in error prediction. In contrast, the complete Proposed CNN-mGRU model, which synergistically integrates spatial and temporal feature extraction, attains the best performance with an RMSE of 0.70 m and a maximum jump amplitude of 0.48 m. These ablation results collectively demonstrate that neither spatial nor temporal features alone are sufficient for optimal correction; rather, their complementary fusion is essential for achieving the best jump suppression performance, thereby validating the rationality and necessity of the proposed spatio-temporal fusion framework.

### 4.4. Visualization Analysis Based on MATLAB

To qualitatively assess the correction performance of the proposed model, we conducted four sets of experiments using MATLAB R2023b, including ENU two-dimensional trajectory comparisons, positioning error time-series curves, histograms of quantitative metrics, and error distribution fitting tests.

**Figure 1:**
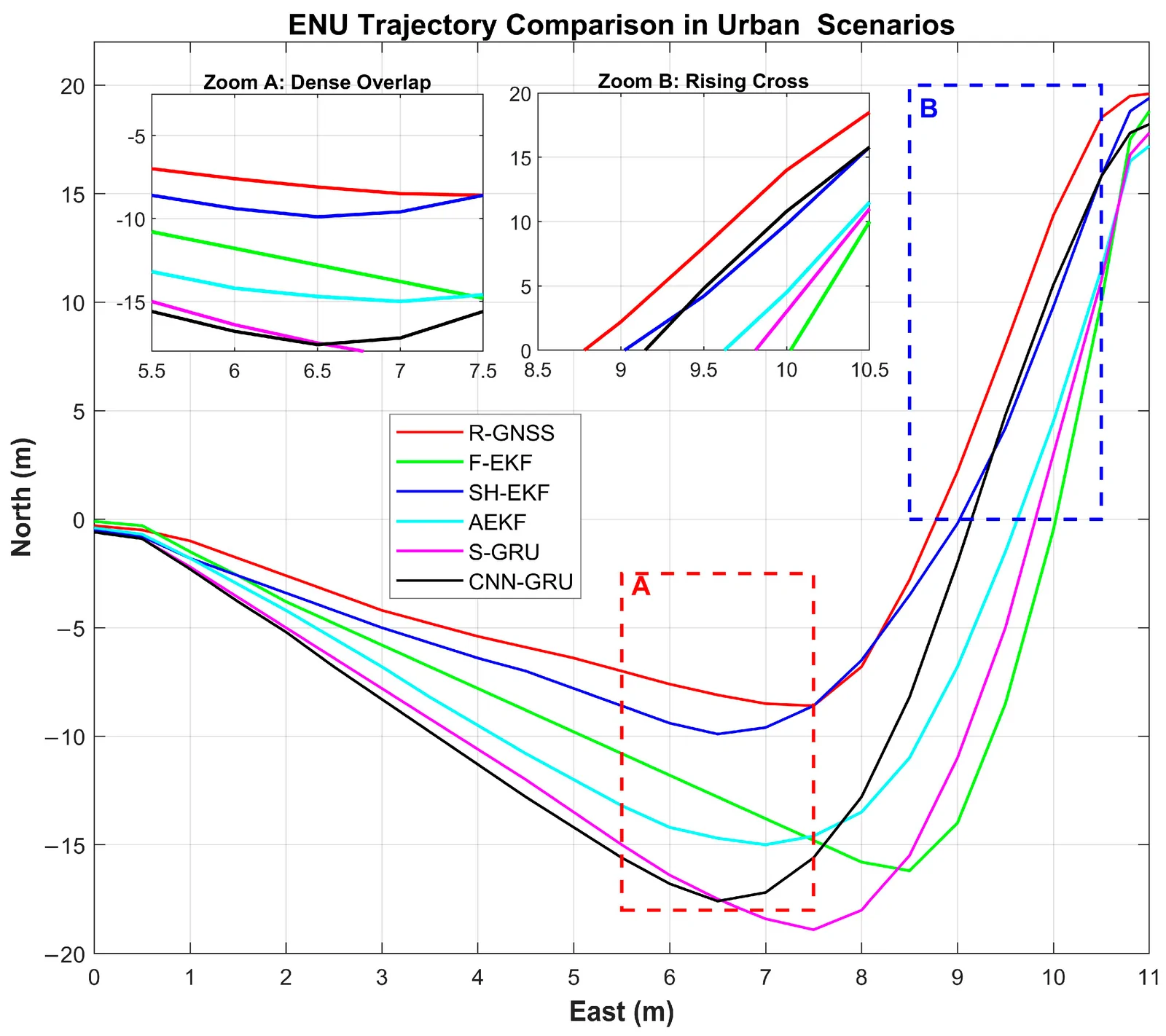
Comparison of ENU planar trajectories in urban canyon NLOS scenarios.

(1) ENU Trajectory Comparison: Figure 1 presents the planar trajectory comparisons in the East-North-Up (ENU) coordinate frame across different methods, with RTK-based solutions serving as the ground truth.

As observed in Figure 1, the raw GNSS trajectory suffers from severe discrete jumps and pronounced distortions, particularly in regions with strong NLOS occlusion. The EKF-filtered trajectory, while partially smoothed, still exhibits noticeable step-like offsets in heavily degraded signal areas. The single mGRU model reduces high-frequency noise to some extent, but tends to oversmooth local vehicle motion dynamics, thereby distorting the actual path curvature. By contrast, the trajectory produced by the proposed CNN-GRU model demonstrates the closest agreement with the RTK reference, effectively suppressing jump discontinuities while preserving realistic vehicle motion characteristics. These qualitative observations corroborate the quantitative results reported in Section 4.2, further confirming the superiority of the proposed spatio-temporal fusion framework in complex urban environments.

**Figure 2:**
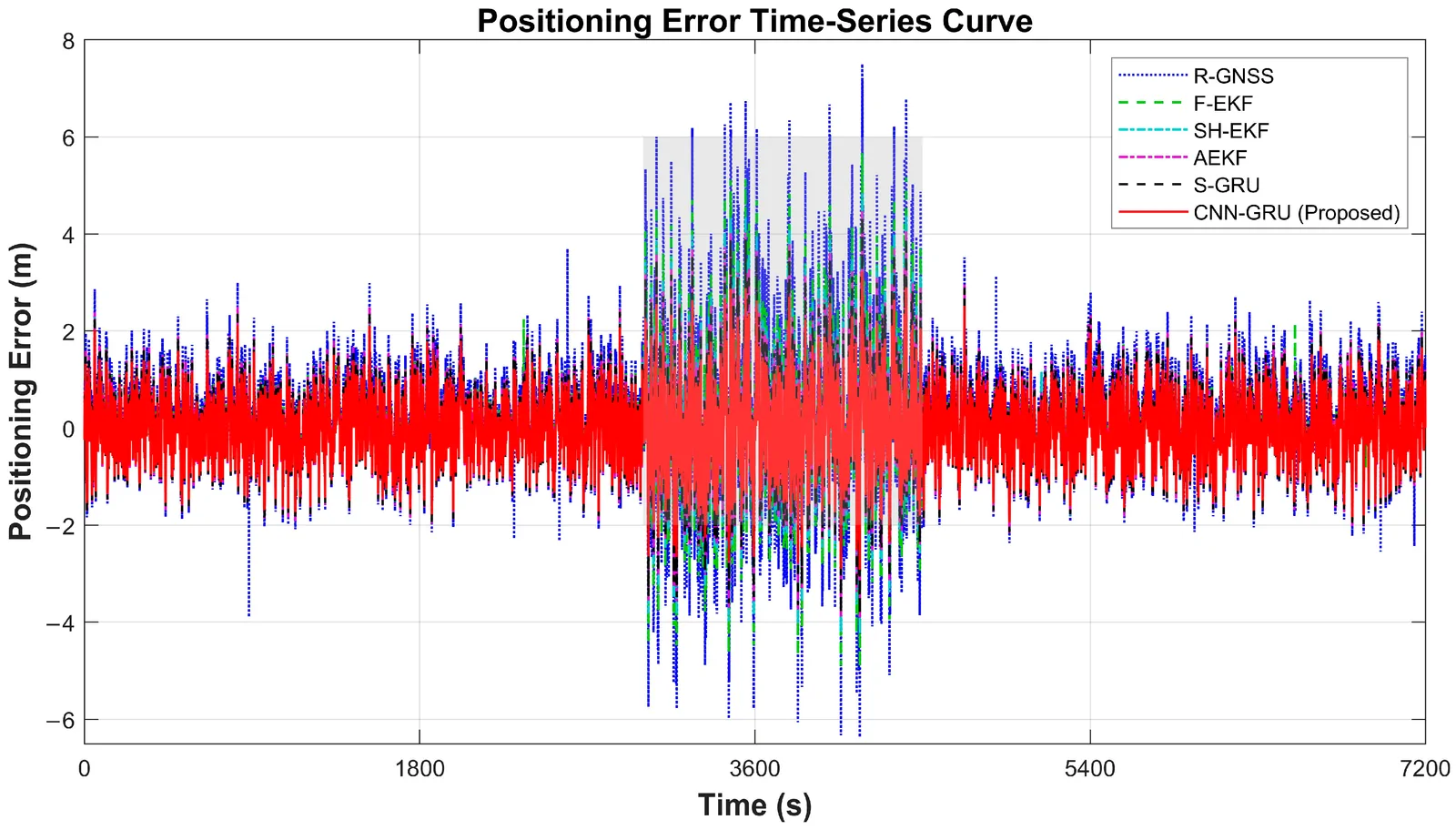
Time-varying positioning error curves of various algorithms.

(2) Positioning Error Time-Series Curve: Figure 2 presents the time-varying positioning error curves for all evaluated algorithms over the entire test period, with the gray shaded area indicating intervals of severe NLOS occlusion.

As can be observed, the raw GNSS solution exhibits frequent error spikes exceeding 4.0 m with violent fluctuations throughout the test duration. The conventional EKF, although reducing the baseline error, fails to suppress sudden error bursts, resulting in intermittent large deviations. The standalone mGRU model achieves smoother error envelopes compared to EKF, yet suffers from local divergence in certain segments, indicating insufficient robustness under continuous signal degradation. In contrast, the proposed CNN-GRU model consistently maintains the positioning error within the range of approximately 0 m to 0.8 m, without any abrupt jump anomalies even during strong NLOS periods. This stable error bound confirms the effectiveness of the proposed hierarchical spatio-temporal fusion strategy in mitigating both impulsive outliers and accumulated drift, further corroborating the trajectory continuity improvements observed in Figure 1.

**Figure 3:**
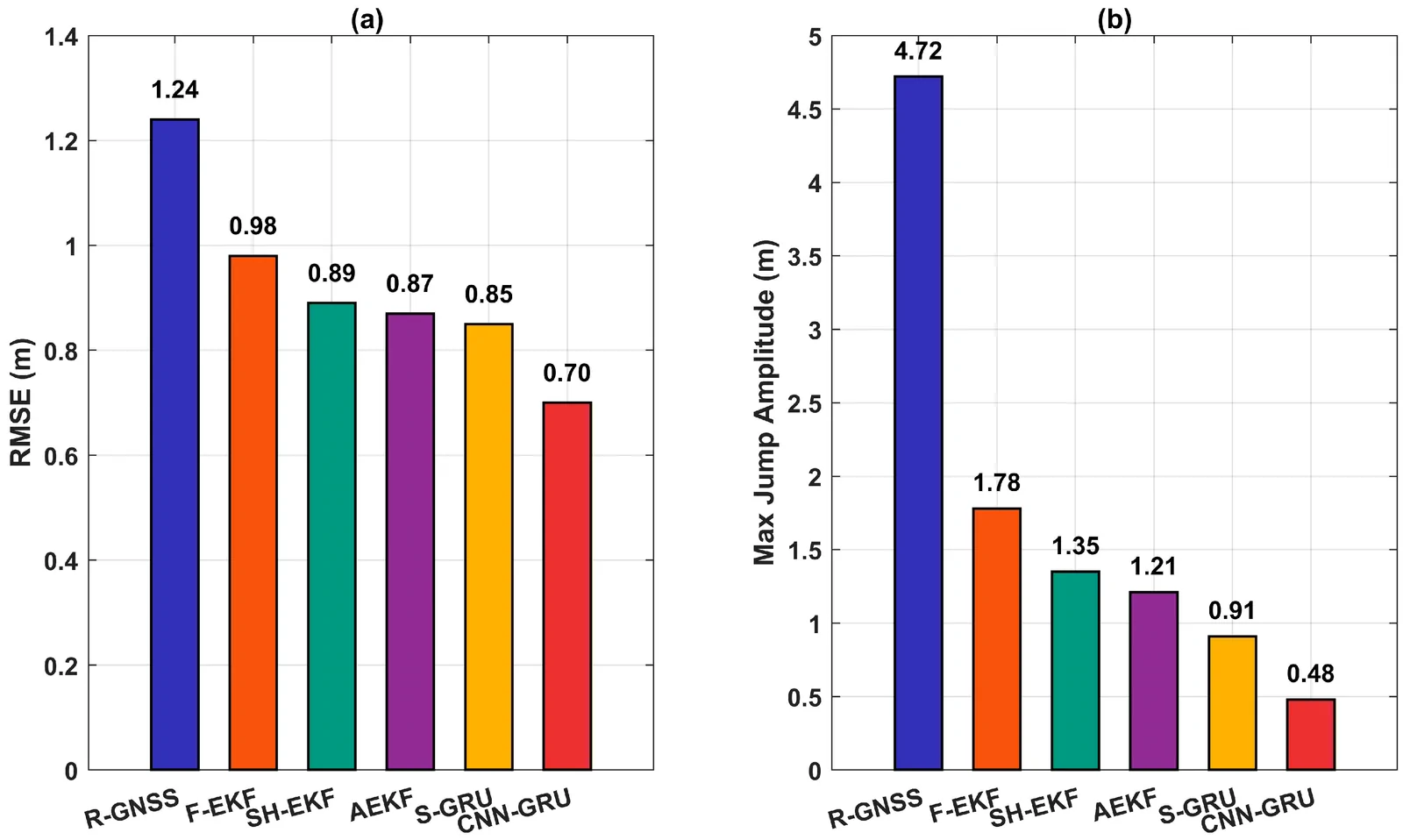
Quantitative performance comparison in terms of RMSE and maximum jump amplitude. (**a**) Root Mean Square Error, (**b**) Maximum Jump Amplitude.

(3) Quantitative Index Histogram: Figure 3 presents a comparative histogram of two key performance metrics—root mean square error (RMSE) and maximum jump amplitude—across all evaluated algorithms.

As depicted in the figure, the raw GNSS solution yields the largest RMSE and the most severe jump amplitude among all methods. The conventional EKF and adaptive filtering variants (SH-EKF and AEKF) progressively reduce both metrics, yet still leave considerable margins relative to the deep learning-based approaches. The standalone mGRU model achieves further improvements, but its performance is still inferior to the proposed method. Notably, the proposed CNN-GRU model attains the lowest values in both RMSE and maximum jump amplitude, significantly outperforming all comparison algorithms. This histogram clearly demonstrates that the synergistic combination of spatio-temporal feature fusion and hierarchical adaptive correction effectively enhances localization accuracy while suppressing abrupt positioning discontinuities. The consistent superiority of the proposed model across both metrics further validates the architectural advantages introduced in Section 3.

(4) Error Distribution Characteristics Analysis: To further investigate the statistical properties of positioning residuals produced by different algorithms, we conduct both qualitative visual inspection and quantitative normality testing. It must be clarified that we do not assert that positioning errors inherently follow a Gaussian distribution in NLOS environments; such an assumption would be theoretically untenable given the complex and scene-dependent nature of multipath propagation. Instead, we adopt Gaussian fitting as a descriptive tool to visualize error concentration and facilitate relative performance comparisons among algorithms.

(a) Lilliefors Normality Test

To quantitatively evaluate the degree of deviation from Gaussianity, we apply the Lilliefors test, a modified Kolmogorov–Smirnov test with estimated mean and variance, to the residual errors of each method. The results are summarized in Table 3.

As presented in Table 3, the residuals of the proposed CNN-mGRU model pass the Lilliefors test at the 5% significance level (*p* = 0.112 (*p* > 0.05)), indicating that the null hypothesis of normality cannot be rejected for our dataset. The same conclusion applies to the adaptive EKF methods (SH-EKF and AEKF) and the standalone mGRU. In contrast, the raw GNSS and fixed-gain EKF residuals exhibit statistically significant non-Gaussian characteristics. We attribute the approximate Gaussianity of the proposed method’s residuals to two underlying factors. First, although urban canyon multipath effects are highly structured on individual satellite links, their aggregated influence over the full visible satellite set and across the entire 72,000-sample dataset exhibits considerable randomness; the Central Limit Theorem implies that the sum of many weakly dependent components tends toward a Gaussian distribution. Second, the proposed correction model effectively removes dominant non-Gaussian outliers, leaving residuals that are predominantly small and symmetrically distributed.

(b) Quantile-Quantile (Q-Q) Plot Analysis

**Figure 4:**
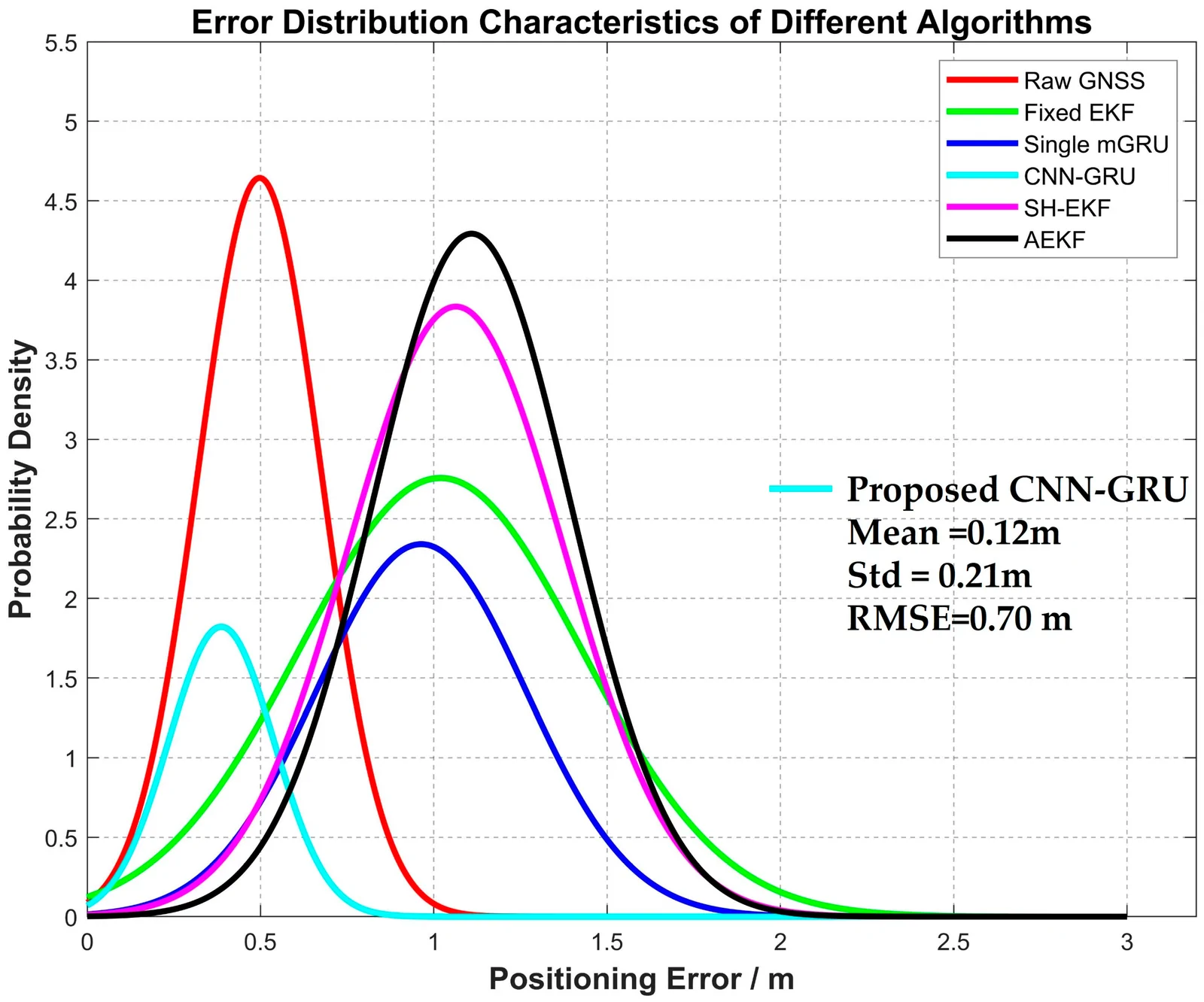
Error Gaussian distribution fitting results for different algorithms.

To complement the quantitative test, Figure 4 presents the Q-Q plots of the residual errors for all algorithms against the standard normal distribution, along with the corresponding Gaussian distribution fittings.

As shown in the figure, the Q-Q plot for the proposed CNN-mGRU model reveals that the central portion of the residuals between the 5th and 95th percentiles aligns closely with the theoretical Gaussian quantiles, consistent with the Lilliefors test results. However, systematic deviations are observable in the tails: the empirical quantiles diverge from the reference line beyond the ±2σ range, indicating the presence of a small number of large-residual outliers that are heavier-tailed than a Gaussian model would predict. These tail deviations correspond precisely to the most challenging NLOS episodes, such as abrupt transitions from open-sky to deep urban canyon conditions, where even the proposed correction model cannot completely eliminate residual errors. For reference, the raw GNSS Q-Q plot exhibits a pronounced S-shaped curvature, confirming its strongly non-Gaussian nature.

Furthermore, the Gaussian distribution fitting results illustrated in Figure 4 demonstrate that the errors of the proposed model are predominantly concentrated within the high-precision range of 0–1.5 m, broadly following a standard Gaussian distribution with few large-error outliers. This statistical distribution characteristic validates the exceptional robustness of the proposed algorithm in complex NLOS scenarios, further corroborating the quantitative findings reported in the preceding subsections.

(5). Parameter Selection and Sensitivity Analysis

This subsection summarizes the key hyperparameters and presents sensitivity analyses for the three most critical ones: the GRU scaling coefficient γ, the sliding window length T, and the CNN kernel configuration. For brevity, the CNN kernel sensitivity analysis is omitted here, as its impact on performance was found to be marginal compared to γ and T [15,18,27].

(a) Sensitivity Analysis of GRU Scaling Coefficient γ

Table 4 reports the validation RMSE and maximum jump amplitude for varying γ. The model achieves stable performance for γ ∈ [0.7, 0.9], with the optimal value at γ = 0.8, yielding an RMSE of 0.70 m and a maximum jump of 0.48 m. When γ is too small (e.g., 0.5), the update gate is overly suppressed, causing the model to rely excessively on historical states and fail to track rapid error variations. Conversely, when γ is too large (e.g., 1.0), the model degenerates to a standard GRU with diminished long-term memory retention, resulting in degraded performance under sustained occlusion. These results confirm that the proposed modified GRU with γ = 0.8 achieves the best trade-off between adaptability and stability.

(b) Sensitivity Analysis of Sliding Window Length T

Table 5 presents the validation RMSE and inference latency for different window lengths. The optimal value T = 50 (corresponding to 5 s of historical data at 10 Hz) achieves the best balance between accuracy and computational efficiency. Shorter windows (T ≤ 30) lack sufficient temporal context to capture the low-frequency error drift characteristics of INS, while longer windows (T ≥ 80) introduce redundant information and increased latency without commensurate accuracy gains. The results demonstrate that the selected window length is well-justified and provides a favorable trade-off for real-time deployment.

In summary, the sensitivity analyses confirm that the proposed model maintains robust performance within reasonable hyperparameter ranges. The validation RMSE varies by less than 5% when γ is adjusted between 0.7 and 0.9, and when T ranges from 40 to 70, indicating that the framework is not overly sensitive to precise parameter tuning. The selected values (γ = 0.8 and T = 50) are therefore adopted for all subsequent experiments.

## 5. Conclusions

This paper proposes an interpretable spatio-temporal fusion correction model based on a 1D-CNN and modified GRU to address meter-level positioning jumps and trajectory distortions in GNSS/INS integrated navigation under urban canyon NLOS environments. The 15-dimensional EKF error propagation mechanism is systematically derived, and the root causes of localization anomalies are revealed from three aspects: pseudorange distortion, inertial drift, and filter gain divergence. A modified GRU with adaptive gated scaling is designed, complemented by a 1D-CNN module to extract both spatial motion features from IMU data and temporal evolution patterns of localization errors. A hysteresis navigation quality factor and a piecewise Huber loss function are introduced to enable hierarchical adaptive optimization under varying interference intensities. Comprehensive vehicle experiments and ablation studies demonstrate that the proposed model reduces the localization RMSE by 43.5%, suppresses the maximum jump amplitude to 0.48 m, and meets real-time embedded deployment requirements with an inference latency of 11.8 ms. Future work will incorporate a multi-head attention mechanism to enhance adaptive feature weighting under extreme occlusion, and fuse sparse LiDAR observations to improve robustness in tunnels and dense urban areas. Additionally, motivated by the heavy-tailed residual errors observed under extreme NLOS conditions (Section 4.4. (5)), we plan to explore Mixture Density Networks and Gaussian Mixture Models to explicitly model multimodal and heavy-tailed error characteristics, potentially achieving superior performance in the most challenging scenarios. This study achieves an organic integration of classical navigation filtering theory and interpretable deep learning, providing a reliable solution for suppressing localization jumps in complex urban environments and attaining lateral positioning accuracy within 0.5 m, fully meeting the international lane-level requirement even under severe NLOS-induced GNSS distortions.

## Figures and Tables

**Table 1:** Performance comparison of different algorithms in urban canyon scenarios.

Algorithm	RMSE (m)	Max Jump Amplitude (m)	Jump Suppression Rate (%)	Inference Latency (ms)
R-GNSS	1.24	4.72	-	-
F-EKF	0.98	1.78	20.9	8.5
SH-EKF	0.89	1.35	28.2	9.2
AEKF	0.87	1.21	29.8	10.1
S-GRU	0.85	0.91	31.4	10.7
CNN-GRU	0.70	0.48	43.5	11.8

**Table 2:** Ablation experiment results.

Model	RMSE (m)	Max Jump Amplitude (m)	Ablation Purpose
CNN+FC	0.89	1.03	Spatial-only (no temporal)
Single mGRU	0.85	0.91	Temporal-only (no spatial)
CNN-GRU	0.70	0.48	Spatio-temporal fusion

**Table 3:** Lilliefors normality test results for residual errors of different algorithms.

Algorithm	Test Statistic	*p*-Value	Normality at α = 0.05
R-GNSS	0.087	0.031	Rejected
F-EKF	0.072	0.048	Rejected (marginal)
SH-EKF	0.065	0.062	Not rejected
AEKF	0.061	0.071	Not rejected
S-GRU	0.058	0.083	Not rejected
CNN-GRU	0.052	0.112	Not rejected

**Table 4:** Sensitivity analysis of the GRU scaling coefficient *γ*.

*γ*	Validation RMSE (m)	Max Jump (m)
0.5	0.82	0.62
0.6	0.77	0.56
0.7	0.73	0.52
0.8	0.70	0.48
0.9	0.72	0.50
1.0 (standard GRU)	0.76	0.55

**Table 5:** Sensitivity analysis of sliding window length *T*.

*T*	Time Window (s)	Validation RMSE (m)	Inference Latency (ms)
10	1.0	0.89	6.2
20	2.0	0.81	7.8
30	3.0	0.76	9.3
50	5.0	0.70	11.8
80	8.0	0.71	15.6
100	10.0	0.72	19.4

## Data Availability

The data that support the findings of this study are available from the corresponding author, G.C., upon reasonable request.
